# Potential value of CT-based comprehensive nomogram in predicting occult lymph node metastasis of esophageal squamous cell paralaryngeal nerves: a two-center study

**DOI:** 10.1186/s12967-024-05217-4

**Published:** 2024-04-30

**Authors:** Ting Xue, Xinyi Wan, Taohu Zhou, Qin Zou, Chao Ma, Jieqiong Chen

**Affiliations:** 1 Department of Radiology, Second Affiliated Hospital of Naval Medical University, No. 415 Fengyang Road, Shanghai, 200003 China; 2 Department of Radiology, Frist Affiliated Hospital of Naval Medical University, No. 168 Changhai Road, Shanghai, 200433 China

**Keywords:** Esophageal squamous cell carcinoma, Para-laryngeal lymph node, Radiomics, Nomogram

## Abstract

**Purpose:**

The aim of this study is to construct a combined model that integrates radiomics, clinical risk factors and machine learning algorithms to predict para-laryngeal lymph node metastasis in esophageal squamous cell carcinoma.

**Methods:**

A retrospective study included 361 patients with esophageal squamous cell carcinoma from 2 centers. Radiomics features were extracted from the computed tomography scans. Logistic regression, k nearest neighbor, multilayer perceptron, light Gradient Boosting Machine, support vector machine, random forest algorithms were used to construct radiomics models. The receiver operating characteristic curve and The Hosmer–Lemeshow test were employed to select the better-performing model. Clinical risk factors were identified through univariate logistic regression analysis and multivariate logistic regression analysis and utilized to develop a clinical model. A combined model was then created by merging radiomics and clinical risk factors. The performance of the models was evaluated using ROC curve analysis, and the clinical value of the models was assessed using decision curve analysis.

**Results:**

A total of 1024 radiomics features were extracted. Among the radiomics models, the KNN model demonstrated the optimal diagnostic capabilities and accuracy, with an area under the curve (AUC) of 0.84 in the training cohort and 0.62 in the internal test cohort. Furthermore, the combined model exhibited an AUC of 0.97 in the training cohort and 0.86 in the internal test cohort.

**Conclusion:**

A clinical-radiomics integrated nomogram can predict occult para-laryngeal lymph node metastasis in esophageal squamous cell carcinoma and provide guidance for personalized treatment.

**Supplementary Information:**

The online version contains supplementary material available at 10.1186/s12967-024-05217-4.

## Highlights

- To investigate the value of CT-based radiomics features in predicting occult lymph node metastasis adjacent to recurrent laryngeal nerve in esophageal squamous cell carcinoma.
- The radiomics nomogram showed better performance than the clinical model to predict occult lymph node metastasis adjacent to recurrent laryngeal nerve in esophageal squamous cell carcinoma.
- The radiomics nomogram demonstrated excellent performance in the training, internal validation, and external validation cohort (AUC, 0.97; AUC, 0.86; AUC, 0.63)

**Supplementary Information:**

The online version contains supplementary material available at 10.1186/s12967-024-05217-4.

## Introduction

China is one of the countries in the world with a high incidence and mortality rate of esophageal cancer, and esophageal cancer is mainly squamous cell carcinoma, accounting for more than 95% [1, 2]. The clinical stage of esophageal cancer patients at the beginning of treatment has an important impact on the selection of treatment strategies and can further affect their survival. At present, the treatment of esophageal squamous cell carcinoma in the intermediate and advanced stages is still a surgical-based integrated treatment. Studies [3, 4] have shown that Sanno lymph node dissection, as a mainstream surgical method for esophageal squamous cell carcinoma, can improve the postoperative survival and the accuracy of pathological staging of patients with esophageal cancer, and reduce the postoperative local recurrence rate of esophageal cancer. However, this surgical method is traumatic, has many complications, prolongs the postoperative hospital stay, and seriously affects the postoperative adjuvant treatment. Therefore, it is very important to choose patients suitable for three-field lymph node dissection. Due to the distribution of recurrent laryngeal nerve-draining lymph nodes along the recurrent laryngeal nerve, which is an important region for esophageal cancer metastasis, it has potential to be used as a sentinel lymph node for cervical lymph node dissection [3]. In addition, recurrent laryngeal nerve injury is also an important adverse prognostic factor after esophageal squamous cell carcinoma surgery. Recurrent laryngeal nerve-draining lymph node dissection can improve the 5-year survival rate of esophageal cancer patients. Nakagawa et al. [4] showed that in the case of upper thoracic esophageal squamous cell carcinoma, third-field lymph node dissection significantly prolonged survival compared with second-field dissection. In addition, Igaki et al. [5] have shown a clear survival benefit with third-field versus second-field dissection in patients with esophageal carcinoma of the lower thoracic segment with metastatic involvement of upper and/or middle mediastinal lymph nodes. Ma et al. [6] also showed that third-field lymph node dissection had a clear advantage over second-field lymph node dissection in terms of 1-, 3-, and 5-year survival rates. However, there is still a lack of effective and non-invasive tools for predicting the metastasis of recurrent laryngeal nerve-draining lymph nodes in esophageal squamous cell carcinoma.

Computed tomography is recommended by the NCCN (National Comprehensive Cancer Network, NCCN) guideline as the preferred imaging examination for esophageal squamous cell carcinoma in clinical practice [7, 8]. It can provide high-resolution images with high tissue contrast, and is currently one of the research hotspots in the field of medical image analysis. CT-based radiomics is not dependent on subjective evaluation by radiologists, and objectively and quantitatively measures the pixels and their arrangement patterns in tumor lesions by extracting high-dimensional image features from tumor lesions, then quantifies the internal lesion information of the tumor. This method realizes real-time, comprehensive, and dynamic capture of tumor heterogeneity through image feature extraction and machine learning technology [9]. Radiomics has been widely studied for its potential in differential diagnosis and prognostic prediction of various cancers due to its high accuracy and availability [10–12]. However, there are few studies on predicting the metastasis of recurrent laryngeal nerve-draining lymph nodes in esophageal squamous cell carcinoma and its impact on patient survival prognosis, and most lack independent external validation.

The aim of this study was to develop and validate a CT-based clinical imaging nomogram that predicts occult lymph node metastasis adjacent to the recurrent laryngeal nerve in patients with esophageal squamous cell carcinoma. This helps to timely individualize treatment for patients suitable for three-field lymph node dissection, thus allowing the patient to have a better survival time.

## Materials and methods

### Patients

**Fig. 1:**
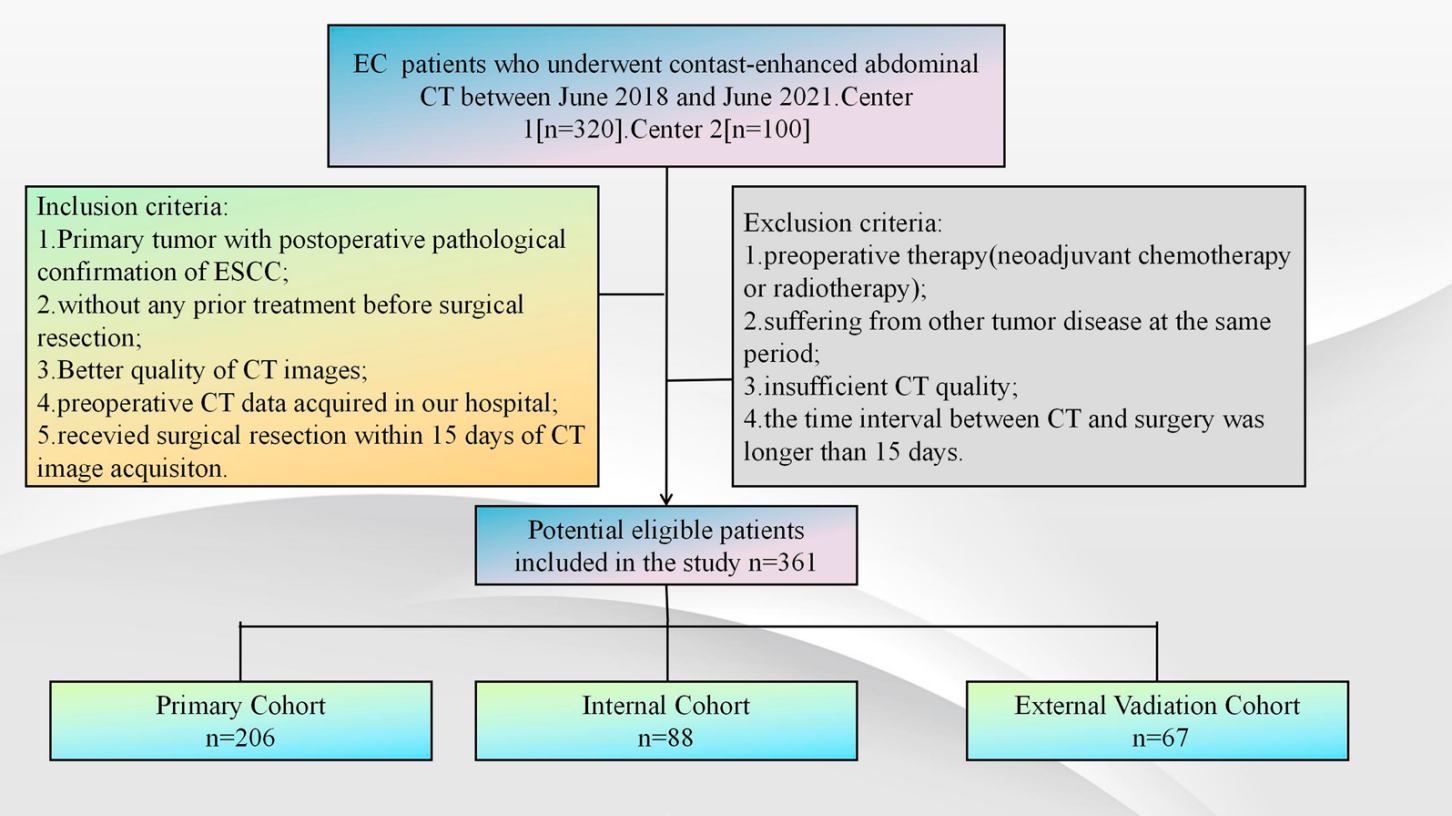
Flow chart of patients’ recruitment pathway

Clinical, pathologic, radiographic, and laboratory data were retrospectively collected from 361 patients with esophageal squamous carcinoma who had undergone surgical pathologic confirmation between May 2015 and December 2017 (294 from center 1; 67 from center 2). Contrast-enhanced CT examination was performed within 1 week before surgery. The present retrospective study gained approval from the institutional review board of our institute. Figure 1 shows the recruitment process. Clinical characteristics of patients including age, gender, smoking history, alcohol consumption history, preoperative carbohydrate antigen199 (CA199), carcinoembryonic antigen (CEA), tumor location, tumor size were obtained from center 1 and center 2. Pathological information included pathological TNM stage, tumor differentiation, lymphovascular invasion (LVI) and perineural invasion (PNI).

Patients conforming to criteria below were included: (1) primary tumor with postoperative pathological confirmation of ESCC; (2) without any prior treatment before surgical resection; (3) better quality of CT images; (4) preoperative CT data acquired in our hospital; (5) received surgical resection within 15 days of CT image acquisition. Patients conforming to criteria below were excluded: (1) preoperative therapy (neoadjuvant chemotherapy or radiotherapy); (2) suffering from other tumor disease at the same period; (3) insufficient CT quality; (4) the time interval between CT and surgery was longer than 15 days.

### CT examination equipment and methods

The patient fasted 4 h before examination and drank 800–1000 mL of water to fill the upper gastrointestinal tract 10–15 min before examination. Mode of scanning: scan in the supine calm breathing state. Scanning Scope: the upper boundary is at the level of the two incisors and the lower boundary is at the level of the lower border of both kidneys. Scan parameters: tube voltage 120 kVP, tube current 100–200 mAs; matrix 512 × 512; layer thickness, layer spacing 5 mm, and 1–2 mm thin layer reconstruction. Arterial phase images were acquired after a CT scan was performed first and then intravenously injected through the elbow median at a rate of 3 mL/s (at 1–1.5 mL/kg) with the contrast agent iohexol (iodine-containing 300 mg/mL, Yangtze River Pharmaceuticals, China) after a delay of 35 s. CT images obtained from scans were uploaded to the image archiving and communication systems (PACS).

According to our discussion with the pathology doctor in the hospital, combined with the scanning range of the chest CT imaging and the new cervical lymph node zoning standard published by the European Society of Radiation Oncology (ESTRO) in November 2023, the observation range of recurrent laryngeal nerve paralymph node metastasis in CT imaging examination is defined as the Vc and VIb area in the cervical lymph node partition. we also provided schematics to illustrate the extent of observation of paralaryngeal lymph nodes that can be detected by CT examination. It is shown in Additional file 1: Fig. S1.

Vc upper and lateral group of clavicle: the upper boundary is the lower edge of the cervical transverse blood vessel, the lower boundary is 2 cm above the upper edge of the sternum handle, the anterior boundary is the skin, the posterior boundary is the anterior edge of the oblus muscle (upper), the anterior front 1 cm (lower) of the anterior saural muscle (lower), the outer boundary is the oblique muscle (upper), the clavicle (lower), the inner boundary is the oblique muscle, the lateral side of the sternum, and the outer side of the IVa area.

VIb prelaryngeal, anterior tracheal and paratracheal lymph nodes: the upper boundary is the lower edge of thyroid cartilage, the lower boundary is the upper edge of the sternum stalk, the anterior boundary is the surface of the throat, the thyroid and trachea (pre-laryngeal and anterior tracheal lymph nodes), anterior vertebral/muscle (right)/esophagus (left), the posterior boundary is the bilateral common carotid artery, and the outside is the side of the trachea and esophagus (lower).

### The detail of pathological confirmation

Postoperative tumor specimens were fixed with 10% formaldehyde, and sections were 4 μm thick and stained for HE. (1) PNI positivity was defined as tumor cell invasion of any layer of the nerve sheath or tumor cell encirclement of at least one-third of the nerve circumference. (2) LVI positivity was defined as the presence of tumor cells within a lumen lined by endothelium, attached to the wall of the tube and with elastic lamellae surrounding the tumor focus. (3) Diagnostic criteria for metastatic involvement of the para-recurrent laryngeal nerve: metastatic involvement of the para-recurrent laryngeal nerve in the specimen submitted was considered to be positive for metastatic involvement of the para-recurrent laryngeal nerve in accordance with the pathologic diagnosis of the esophageal lesion (all specimens were confirmed by the same group of our departmental physicians).

### Image segmentation and feature extraction

**Fig. 2:**
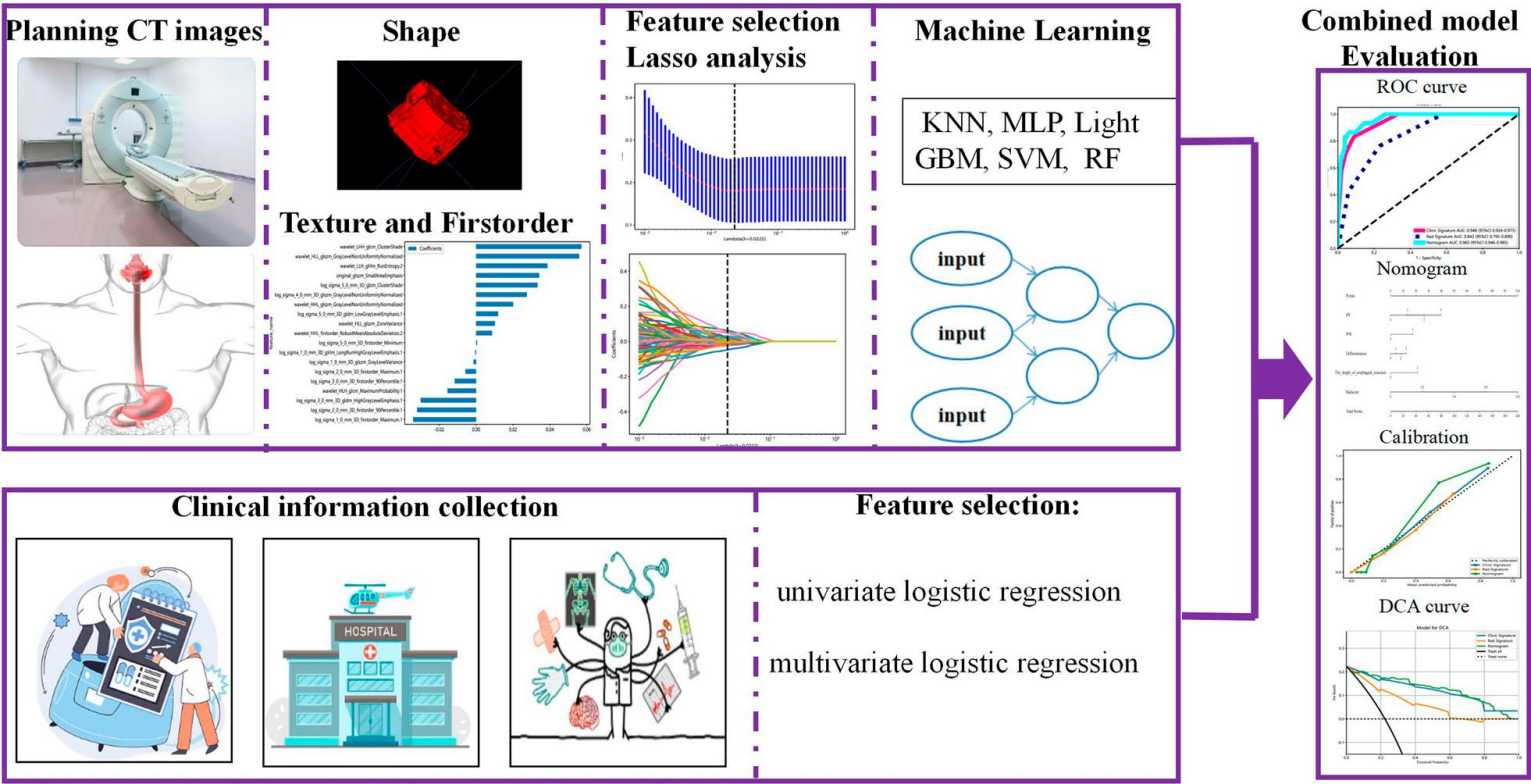
Workflow of this study

The workflow of radiomics is illustrated in Fig. 2. The entire tumor was manually delineated with ITK-SNAP (v. 3.8.0, https://www.itksnap.org) for the period of unenhanced, arterial, and venous phase. Interclass and intraclass correlation coefficients (ICCs) are used to evaluate the interobserver and intraobserver reproducibility of extracted radiohistological features. In this study, CT images of 30 patients randomly selected from the training group were subjected to ROI delineation to calculate the inter- and intra-class correlation coefficients (ICCs) of the extracted radiographic characteristics to assess the intergroup and intra-group agreement of the characteristics extracted. ICCs > 0.75 indicated a better consistency in characteristic extraction.

Prior to extracting radiomics features, three steps were utilized for image preprocessing. Firstly, linear interpolation was utilized for image resampling to 1 mm * 1 mm * 1 mm. Secondly, gray level discretization was applied in converting serial images in the discrete integer values. At last, mixed noise during image digitization was removed using log and wavelet image filters, while high- or low-frequency features were then obtained. Radiomics features were extracted using the open-source package Py Radiomics (version 3.0.1, https://pyradiomics.readthedocs.io/en/latest/). From the unenhanced, arterial, and venous 1024 radiomics features were extracted respectively for quantification of tumor internal heterogeneity. These signatures consisted of 18 First order, 22 grey-level co-occurrence matrix (GLCM), 16 grey-level run-length matrix (GLRLM), 16 grey-level size zone matrix (GLSZM), 14 grey-level dependence matrix (GLDM), and 14 shapes.

### Radiomics features selection and model establishment

The optimal radiomics features were selected by the following three steps. First, a robust radiomics signature of ICCs > 0.75 was selected. Secondly, the mRMR algorithm, LASSO logistic regression, and tenfold cross-validation were used to characterize dimensional reduction. mRMR minimizes the discrimination between negative and positive features of lymph node metastasis in the recurrent laryngeal nerve and eliminates redundant and unrelated features, improving the efficiency of late modeling and modeling by reducing dimensions. Subsequently, LASSO-Logistic regression algorithm and tenfold cross-validation of regulatory punishment parameters (Lambda, λ) were used to select the optimal feature with non-zero coefficients. Finally, a logistic regression model was constructed by calculating Rad-scores for the sum of the respective LASSO-Logistic regression coefficient-weighted selected characteristics with the respective corresponding coefficient product. The predictive power of the imaging histology model was assessed by ROC curves, with AUC, accuracy, sensitivity, and specificity calculated. One hundred LGOCVs were used to test the stability and reliability of the results. DCA was used to evaluate the clinical utility of the model. The selection of all radiomics features and the construction of the models were performed in the training group.

### Establishment of the comprehensive nomogram

Significant clinical predictors were determined by the chi-square test (classified variables), t-test, or Mann–Whitney U-test (continuous variables) in the training group; then univariate logistic regression was used to determine whether these clinical predictors were statistically significant (P < 0.05) and multivariate logistic regression was used to construct a clinical model. To establish a more robust predictive model, imaging integrated nomograms were constructed combining these clinical predictors with imaging scores. Calibration curves for histological nomograms were plotted using the Hosmer–Lemeshow (HL) test. Finally, the net benefit of the decision curve evaluation nomogram was plotted. Statistical analysis Data processing and modeling used R software (v. 4.2.1, https://www.R-project.org). The R-software package used is shown in Table 2. The tests for continuous variables used independent sample t test or Mann–Whitney U test. Categorical variables were tested with the χ^2^ test or Fisher’s exact test. The diagnostic efficiency of each model was evaluated with the use of receiver operating characteristic (ROC) curves.

## Results

### Clinical characteristics

In total, 361 patients with esophageal squamous cell carcinoma were included in the analysis. Tables 1, 2 summarizes the characteristics of the clinical baseline data of this study. In the training cohort, gender, age, smoking, alcohol consumption, tumor location, and tube wall thickness were not statistically significant between patients in the positive and negative group for recurrent laryngeal nerve paralymph node metastasis (both P values > 0.05). The length of pathological N stage, peripheral nerve infiltration, differentiation, and esophagectomy between patients in the positive group and those in the negative group of recurrent laryngeal nerve paralymph node metastasis was statistically significant (P value < 0.05).

**Table 1:** The differences of clinical data in both the training and test cohorts

Characteristic	Training cohort (n = 206)			Internal validation cohort (n = 88)			External validation cohort (n = 67)		
	LNM− group (n = 155)	LNM+ group (n = 51)	p-value	LNM− group (n = 73)	LNM+ group (n = 15)	p-value	LNM− group (n = 44)	LNM+ group (n = 23)	p-value
Sex (%)			0.867			0.024			0.788
Female	37 (23.9)	13 (25.5)		12 (16.4)	7 (46.7)		20 (45.5)	10 (43.5)	
Male	118 (76.1)	38 (74.5)		61 (83.6)	8 (53.3)		24 (54.5)	13 (56.5)	
Age (mean ± SD, years)	62.8 ± 12.2	65.3 ± 8.9	0.654	60.3 ± 13.3	63.5 ± 10.3	0.707	58.3 ± 11.2	64.2 ± 10.8	0.025
Drinking history			0.084			0.128			0.192
No	96 (61.9)	39 (76.5)		40 (54.8)	12 (80.0)		15 (34.1)	4 (17.3)	
Yes	59 (38.1)	12 (23.5)		33 (45.2)	3 (20.0)		29 (65.9)	19 (82.7)	
Smoking history			0.249			0.058			0.107
No	84 (54.2)	33 (64.7)		31 (42.5)	11 (73.3)		18 (40.9)	12 (36.4)	
Yes	71 (45.8)	18 (35.3)		42 (57.5)	4 (26.7)		26 (59.1)	11 (63.6)	
Thick (mean ± SD, cm)	1.3 ± 0.3	1.4 ± 0.4	0.054	1.4 ± 0.6	1.5 ± 0.9	0.907	1.1 ± 0.9	1.0 ± 1.8	0.752
Tumor location (%)			0.934			0.618			0.258
Upper lesion	7 (4.5)	2 (3.9)		3 (4.1)	1 (6.7)		15 (34.1)	10 (43.5)	
Lower lesion	99 (63.9)	34 (66.7)		53 (72.6)	9 (60.0)		12 (27.3)	8 (34.8)	
Middle lesion	49 (31.6)	15 (29.4)		17 (23.3)	5 (33.3)		17 (38.6)	5 (21.7)	
pT stage (%)			0.157			0.197			0.927
pT1 + T2	34 (21.9)	6 (11.8)		21 (28.8)	1 (6.7)		10 (22.7)	3 (13.0)	
pT3	107 (69.0)	37 (72.5)		48 (65.8)	13 (86.7)		20 (45.5)	10 (43.5)	
pT4	14 (9.0)	8 (15.7)		4 (5.5)	1 (6.7)		14 (31.8)	10 (43.5)	
pN stage (%)			0.015			0.033			0.886
pN0	14 (9.0)	11 (21.6)		37 (50.7)	3 (20.0)		12 (27.3)	8 (34.8)	
pN1	42 (27.1)	16 (31.4)		25 (34.2)	6 (40.0)		10 (22.7)	12 (52.2)	
pN2	22 (14.2)	12 (23.5)		9 (12.3)	6 (40.0)		12 (27.3)	2 (8.7)	
pN3	7 (4.5)	12 (23.5)		2 (2.7)	0 (0.0)		10 (22.7)	1 (4.3)	
PNI			0.001			0.056			0.228
No	124 (80.0)	25 (49.0)		16 (21.9)	5 (33.3)		20 (45.5)	10 (43.5)	
Yes	31 (20.0)	26 (51.0)		57 (78.1)	10 (66.7)		24 (54.5)	13 (56.5)	
Differentiation			0.014			0.045			0.928
0	42 (27.1)	14 (27.5)		16 (21.9)	5 (33.3)		12 (27.3)	12 (52.2)	
1	113 (72.9)	30 (58.8)		57 (78.1)	10 (66.7)		20 (45.5)	10 (43.5)	
2	0 (0.0)	4 (7.8)		0 (0.0)	0 (0.0)		4 (9.1)	1 (4.3)	
3	0 (0.0)	3 (5.9)		0 (0.0)	0 (0.0)		8 (18.2)	0 (0.0)	
The length of esophageal resection			0.025			0.048			0.128
≤ 5 cm	40 (25.8)	6 (11.8)		13 (17.8)	1 (6.7)		20 (45.5)	13 (56.5)	
> 5 cm	115 (74.2)	45 (88.2)		60 (82.2)	14 (93.3)		24 (54.5)	10 (43.5)

**Table 2:** Univariate and multivariable logistic regression analyses for selecting clinical data of model development

Variable	Univariate analysis		Multivariate analysis	
	OR (95% CI)	p-value	OR (95% CI)	p-value
pN stage	1.15 (1.11–1.20)	0.015	1.02 (0.95–1.10)	0.024
PNI	1.23 (1.12–1.34)	0.001	1.02 (0.95–1.10)	< 0.001
The_length_of_esophageal_resection	1.15 (1.04–1.27)	0.025	1.06 (0.98–1.13)	0.023
Differentiation	1.12 (1.04–1.21)	0.014	1.06 (1.01–1.13)	0.045
Rad score	6.91 (3.15–9.29)	< 0.001	7.86 (2.82–21.95)	< 0.001

### Features selection and radiomics model establishment

**Fig. 3:**
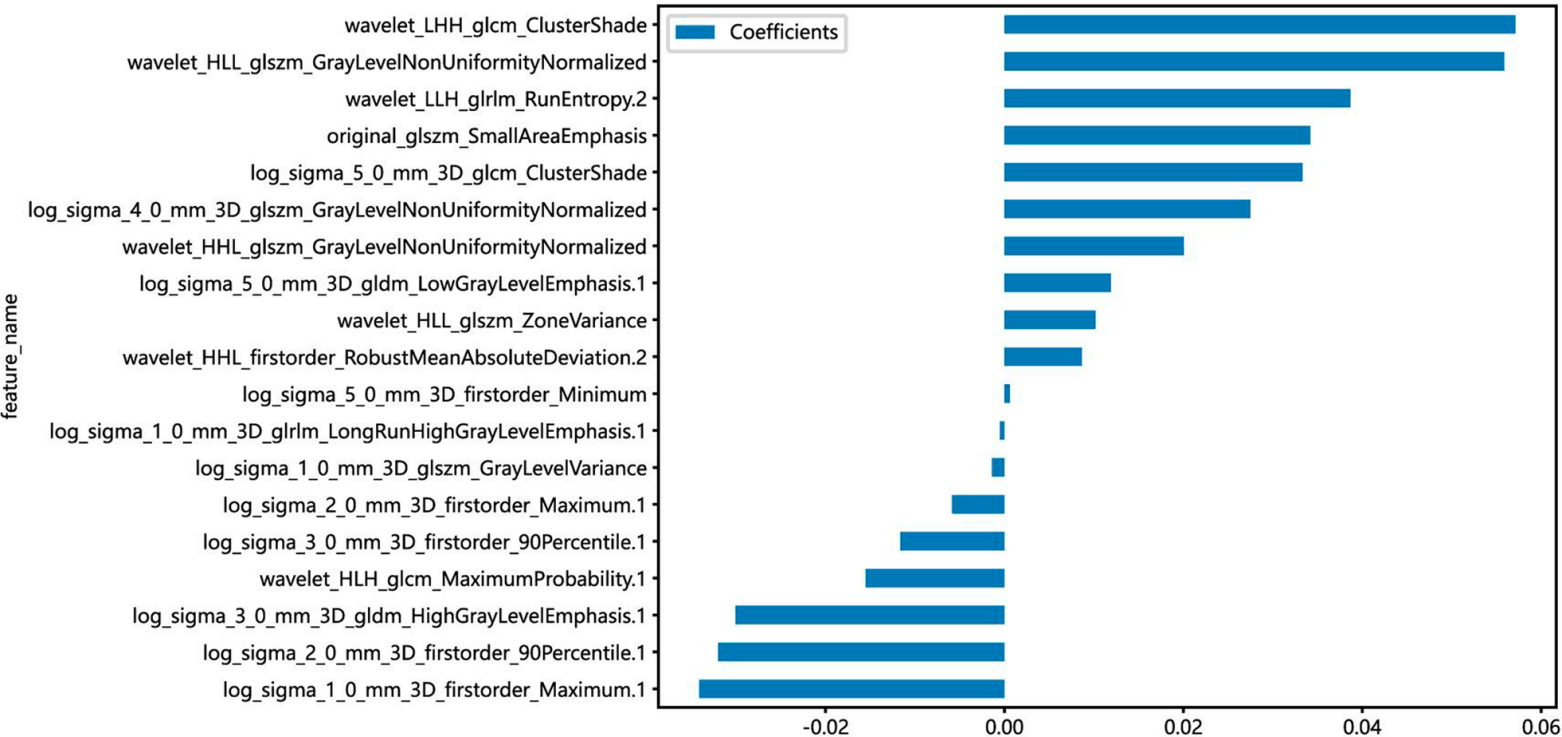
The 19 optimal radiomic features selected for the radiomics model are shown in this image, illustrating the contribution of each feature in the radiomics model

First, 1024 radioimaging histological features (ICCs > 0.75) were retained. Secondly, the mRMR algorithm was used to reduce the number of signatures to 30. Finally, the LASSO regression with an optimal λ of 0.022 determined the imaging histology characteristics of 20 non-zero coefficients. Additional file 1: Fig. S2 showed that lasso was used to develop Radscore. Additionally, this study compared the stability and reliability of five machine learning methods with logistic regression models constructed to predict recurrent paraneural lymph node metastasis in esophageal squamous cell carcinoma, including k nearest neighbor (KNN), multilayer perceptron (MLP), light Gradient Boosting Machine (Light GBM), support vector machine (SVM), random forest (RF), and calculated AUC, accuracy, sensitivity, and specificity (Table 3 and Fig. 3). The results showed that the imaging histology model constructed with the KNN method had the best stability and reliability. The KNN-based radiomics model was finally selected for the establishment of subsequent comprehensive nomogram.

**Table 3:** Diagnostic performance of different radiomics models for predicting osteoporosis in training and test cohorts

Model	Accuracy	AUC	95% CI	Sensitivity	Specificity	PPV	NPV
LR-train	0.83	0.78	0.71–0.85	0.37	0.97	0.78	0.84
LR-test	0.86	0.86	0.72–1.00	0.57	0.95	0.79	0.87
SVM-train	0.83	0.95	0.92–0.99	0.27	0.99	0.99	0.82
SVM-test	0.80	0.81	0.63–1.00	0.14	0.99	0.99	0.79
KNN-train	0.82	0.84	0.79–0.89	0.72	0.94	0.67	0.85
KNN-test	0.81	0.62	0.37–0.86	0.74	0.95	0.68	0.78
Random Forest-train	0.98	0.99	0.99–1.00	0.93	0.99	0.99	0.98
Random Forest-test	0.76	0.73	0.55–0.92	0.54	0.95	0.49	0.78
Light GBM-train	0.80	0.97	0.95–0.98	0.53	0.99	0.88	0.79
Light GBM-test	0.76	0.76	0.51–1.00	0.50	0.99	0.49	0.76
MLP-train	0.81	0.83	0.78–0.89	0.16	0.99	0.90	0.8
MLP-test	0.76	0.83	0.67–0.98	0.50	0.99	0.50	0.76

### Establishment and performance of the clinical model

Patients from the training cohort were used to construct the prediction model. Clinical and pathological information of 206 patients with esophageal cancer results of the univariate logistic regression and multivariate logistic regression analyses are shown in Tables 1, 2. The results of univariate logistic regression showed that the 3 parameters of lesion differentiation, pathologic N stage, and the presence or absence of peripheral nerve invasion (P < 0.05) significantly contributed to the prediction of recurrent laryngeal paralymph node metastasis in esophageal cancer and were included in the multivariate logistic regression analysis. The results of multivariate logistic regression analysis demonstrated that the 3 parameters of lesion differentiation, pathologic N stage, and the presence or absence of peripheral nerve invasion (P < 0.05) were identified as risk factors of differential diagnostic value and incorporated to construct a clinical model. The predictive accuracy of clinical models is suboptimal. The AUC was 0.94 [95% CI 0.92–0.97] with accuracy of 67% in the training set and 0.85 (95% CI 0.67–1.00) with accuracy of 90% in the internal validation set (Table 4).

**Table 4:** Diagnostic performance of better radiomics, clinical and combined models for predicting osteoporosis in training and test cohorts

Model	Cohort	AUC (95% CI)	Accuracy	Sensitivity	Specificity	PPV	NPV
Radiomics mode	Training cohort	0.84 (0.79–0.89)	0.83	0.73	0.94	0.68	0.85
	Internal validation cohort	0.62 (0.37–0.86)	0.77	0.64	0.95	0.50	0.79
	External validation cohort	0.63 (0.49–0.78)	0.65	0.17	0.70	0.24	0.62
Clinical model	Training cohort	0.94 (0.92–0.97)	0.67	0.62	0.70	0.55	0.75
	Internal validation cohort	0.85 (0.67–1.00)	0.90	0.57	0.99	0.99	0.88
	External validation cohort	0.59 (0.44–0.73)	0.68	0.26	0.73	0.33	0.65
Combined model	Training cohort	0.97 (0.95–0.99)	0.91	0.68	0.98	0.89	0.91
	Internal validation cohort	0.86 (0.67–1.00)	0.80	0.72	0.99	0.99	0.79
	External validation cohort	0.63 (0.47–0.78)	0.70	0.77	0.22	0.65	0.33

### Establishment and performance of the comprehensive nomogram

**Fig. 4:**
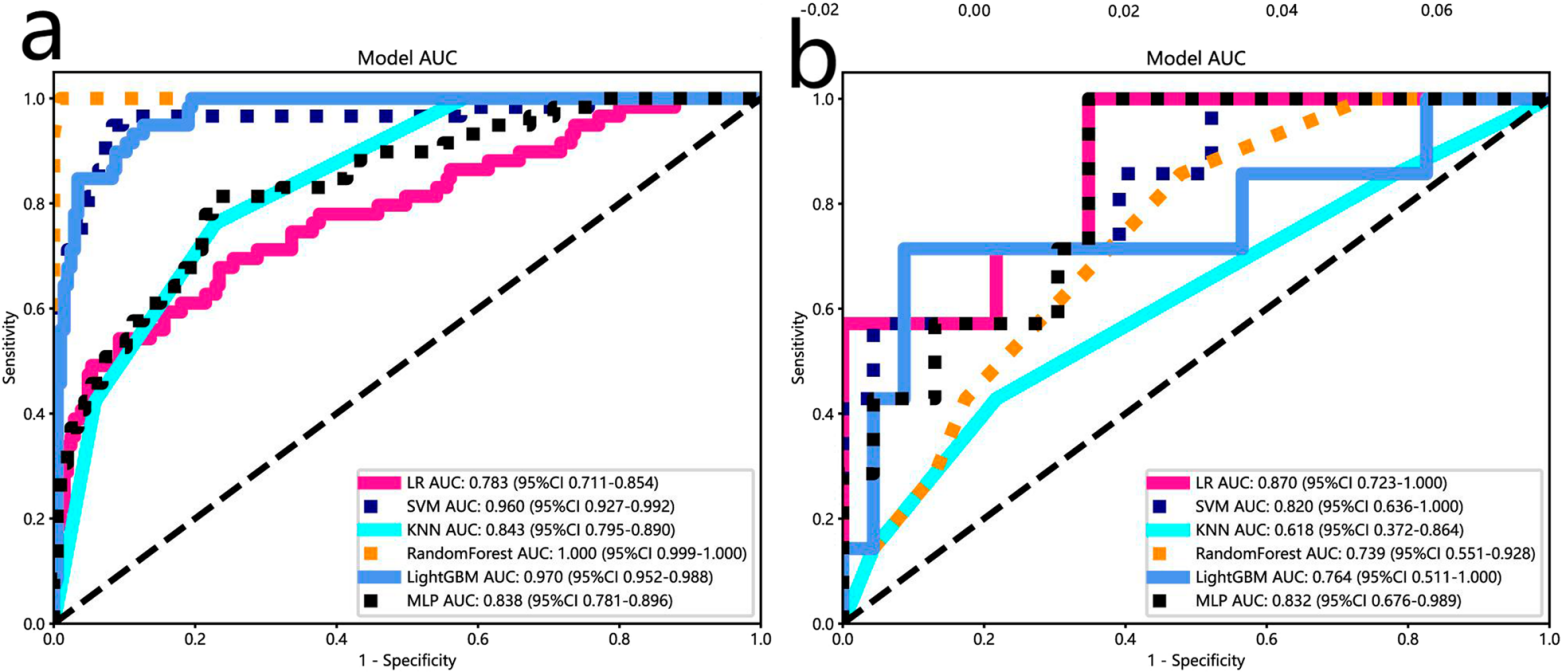
The receiver operating characteristic curves for the radiomics models (LR, SVM, RF, KNN, MLP) in the training (**a**) and test (**b**) cohorts are shown. The y-axis represents the true positive rate (sensitivity), and the x-axis represents the false positive rate (1-specifcity). The diagonal line represents the performance of a random classifier

**Fig. 5:**
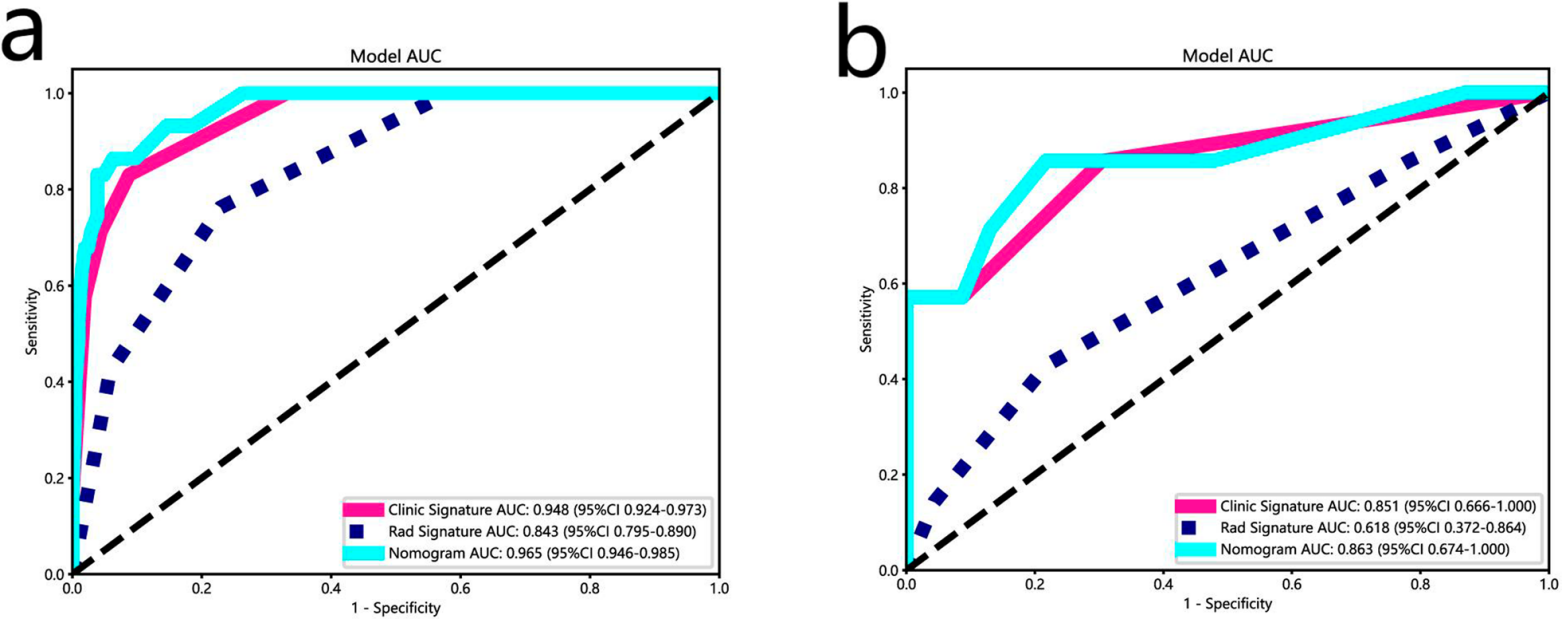
The receiver operating characteristic curves of the clinical, radiomics, and combined models in the training (**a**) and test (**b**) cohorts are displayed. It is plotted with the true positive rate (sensitivity) on the y-axis against the false positive rate (1-specifcity) on the x-axis. The diagonal line represents the performance of a random classifier. Rad radiomics model (KNN); *AUC* area under the curve

**Fig. 6:**
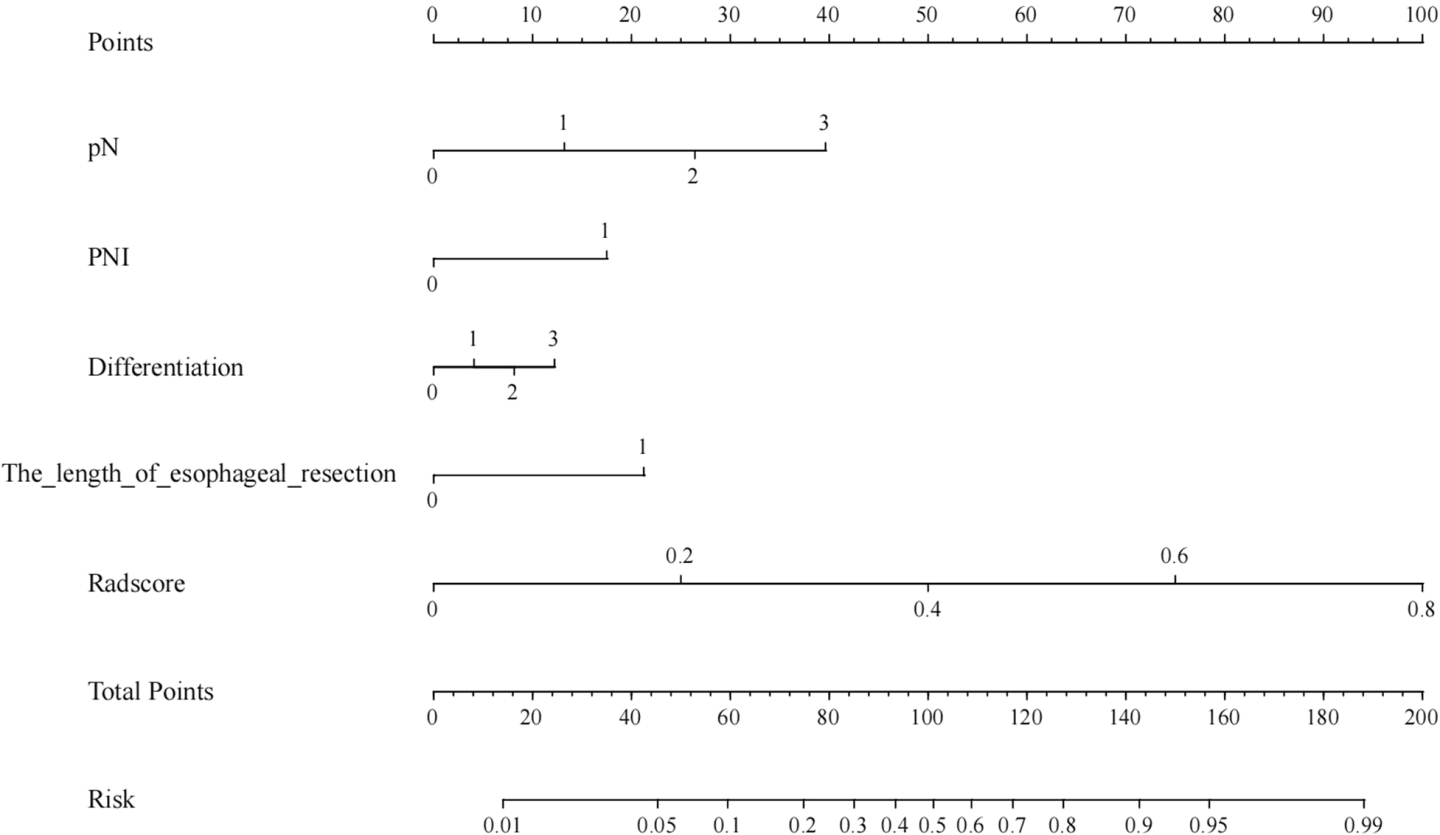
Comprehensive nomogram for predicting recurrent laryngeal nerve paralymph node metastasis in esophageal squamous carcinoma

In this study, clinical factors were analyzed using univariate and multivariate logistic regression, which showed that pathological N stage, peripheral nerve infiltration, differentiation, and length of esophagectomy were statistically significant for predicting recurrent laryngeal nerve paralymph node metastasis in esophageal squamous cell carcinoma in the training group (P < 0.05) (Tables 1, 2). Therefore, a clinical model was constructed according to clinical risk factors, and the efficacy of the clinical model is shown in Table 3. Clinical predictors were finally combined with a KNN-based rad-score with high stability and reliability (Table 4 and Figs. 4, 5) to construct the integrated nomogram in Fig. 6 and Additional file 1: Figs. S3 and S6. The HL (Hosmer–Lemeshow, HL) test showed good agreement (P values > 0.05) for the calibration curves of the nomograms predicting recurrent laryngeal nerve paralymph node metastases in both the training and validation groups, as shown in Additional file 1: Fig. S4.

Decision curve analysis (DCA) can help to judge the actual clinical utility of clinical model, radiomics model, combined model. The decision curves for both models suggest that this new diagnostic strategy results in a greater net benefit (> 0 means patient benefit) in patients with recurrent laryngeal nerve occult lymph node metastases, with a more pronounced benefit in the clinical-radiomics model than in the clinical model, as shown in the Additional file 1: Fig. S5.

## Discussion

This study establishes and validates the predictive value of radiomics models based on preoperative contrast-enhanced CT images for laryngeal recurrent occult lymph node metastasis in esophageal squamous carcinoma. Additionally, comprehensive nomograms based on rad score and clinical predictors (pathological N stage, peripheral nerve infiltration, length of esophagectomy, and degree of differentiation) were most predictive.

Detecting lymph node metastasis in routine CT examination is extremely challenging. Particularly metastatic lymph nodes less than 1 cm in diameter [13, 14]. Although routine CT examination is convenient and popular, the diagnostic performance of lymph node metastasis is relatively low. Previous studies [15, 16] have shown that imaging based on CT, MRI, PET-CT, etc., as a noninvasive and quantifiable method, not only allows observation of the anatomical structure of the tumor, but also reflects tumor heterogeneity and has good predictive accuracy in multiple tumor nodal status predictions. In this study, we found that in addition to texture characteristics, first-order characteristics are also of great value in the optimal model, with a total of 6 so-called first-order characteristics, which refer to the differences and patterns in the distribution of pixel gray intensity in raw data images directly based on CT scans, used to describe the distribution of signal intensity worthy of various voxels, which can be reflected by the distribution characteristics of histograms [17, 18]. Thus, the difference in gray-value intensity caused in the CT images within the tumor tumor was of great significance for the model predicting lymph node metastasis with para-laryngeal nerve occultness. In addition, we found that the correlation coefficients of GLCM, GLDM, GLRLM, and GLSZM imaging characteristics ranked fourth. Among these selected 3D imaging characteristics, Cluster Shade based on the gray-level symbiotic matrix of 3D images was the most valuable parameter for predicting recurrent laryngeal nerve lymph node metastasis in esophageal squamous carcinoma. The cluster shading reflects the uniformity and equilibrium of the gray matter value of the image, i.e., the larger the local change in the image texture, the larger the texture heterogeneity, the larger the cluster shading value.

This study is based on manually sketching the CT image of the area of interest, which reflects the heterogeneity of the tumor in the microstructure and improves the predictive performance of the model. This study uses ITK-SNAP software to extract the histogram parameters, absolute gradient model, grayscale travel matrix and grayscale symbiotic matrix characteristics of the three-dimensional space of the whole tumor, which can reflect the heterogeneity of the whole tumor. Select the imaging omics characteristics with the greatest weight of differential diagnosis. The constructed Radscore has high predictive value. In the training and test set, Radscore in the positive paralaryngeal lymphatic metastasis is higher than the negative lymph node metastasis group, indicating that the esophageal cancer lymph node metastasis group and the non-metastatic group have no For the heterogeneity of the same tumor, the AUC, which uses the KNN-based radiomics model alone, is 0.881 and 0.741 in the training and test set, which has high efficiency, indicating that the radiomics feature predicts that the case of lymph node metastasis that is negative by CT images suggests has potential value, high Rad-scores suggests that the possibility of lymph node metastasis is significantly increased.

This study also compared several machine learning approaches to differentiate preoperative recurrent laryngeal nerve lymph node status in patients with esophageal squamous cell carcinoma and found that the KNN machine learning model had the best predictive efficacy, with AUC values > or = 0.80 in both the training and test sets, and other machine learning models, but the limitations of the algorithm and the loss of important clinical observational characteristics prevented it from comparing with the KNN model. Therefore, the KNN machine learning model constructed in this study has high practicality and reliability. The reason why KNN machine learning models achieved the expected diagnostic efficiency may be as follows: first, the theoretical maturity and simplicity of the KNN algorithm can be used to construct regression as well as linear, nonlinear classification models. Second, compared to machine algorithms such as Basque Bayesian, the KNN algorithm has low complexity, high accuracy, and is insensitive to abnormalities. Third, as the KNN approach relies mainly on the surrounding limited adjacent samples rather than the discriminant-domain approach to determine the class to which it belongs, the KNN algorithm is more appropriate than other approaches for sets of unclassified samples with more crossovers or duplications of the class. Forth, the KNN algorithm compares automatically classifications applicable to categories with larger sample sizes, whereas those with smaller sample sizes tend to be subject to misclassification with this algorithm. Also the reason why other machine learning models failed to achieve the expected diagnostic efficiency may be as follows: first, the small sample size of the present study and the modeling of other machine learning models was simpler than that of the KNN machine learning model. Therefore, they are not applicable to small data volumes. Second, the relationship of CT imaging histological features of esophageal squamous carcinoma to recurrent laryngeal nerve paralymph node status is unclear and is likely to be nonlinear. The KNN machine learning model is more explanatory and suitable for solving a series of complex nonlinear linear problems.

Some scholars have also found [19] that the size of the primary tumour is not a determinant of lymph node metastasis for lymph node metastasis. Similar to this conclusion, in this study we found that none of the final model-selected features were shaped. This suggests that three-dimensional morphological and size characteristics of esophageal squamous carcinoma tumors are not decisive factors in the prediction of lymph node status, and that esophageal squamous carcinoma recurrent para-neural lymph node status may be more dependent on the degree of differentiation, pathological type, and progression [20] of the tumor.

In recent years, more and more studies have developed nomograms to help clinical decision-making processes intuitively, making treatment strategies more precise and personalized for patients with cancer. In previous studies of other tumors, it was found that the imaging histological characteristics of the primary tumor could evaluate and predict lymph node metastasis of gastric adenocarcinoma, lung cancer, bladder cancer and other tumors. At the time of the deadline, the authors searched Pubmed with the MeSH subject-matter ‘recurrent paranodal lymph node metastases in esophageal cancer’ and the keyword ‘Radiomics’, but did not retrieve relevant literature that predicted recurrent paranodal lymph node metastases by radiomics features of primary esophageal cancer. However, metastatic involvement of lymph nodes adjacent to the recurrent laryngeal nerve in esophageal cancer has some commonalities with axillary lymph nodes in breast cancer, i.e., no accurate localization of metastatic lymph nodes can be achieved in both cases.

Therefore, the related study of using imaging histology to predict axillary lymph node metastasis in breast cancer is of great reference significance for laryngeal recurrent nerve paralymph node metastasis in esophageal cancer. Yu et al. [21] developed a nomogram with imaging histological features and clinical features to provide individualized prediction of the risk of axillary lymph node metastasis and disease recurrence in patients with early breast cancer. Tan et al. [22] established nomograms (AUC = 0.805) containing clinical-pathological features of radiohistology based on T2-FS images using linear regression models. Shan et al. [23] validated a nomogram model for invasive detection of axillary lymph node metastasis in patients with breast cancer by combining a kinetic curve model and extraction of imaging histology features from DCE-MRI. These nomograms are based on the analysis of imaging characteristics of breast tumors, and although they are significant in predicting axillary lymph node metastasis, they do not accurately localize metastatic lymph nodes. This is similar to the subject matter of the present study. The nomogram developed in this study was composed of imaging histological features, with Rad-score being the most significant independent influencing factor in differentiating axillary lymph node metastatic status (OR = 7.86, P < 0.001). After adding Rad-score to the prediction model for imaging features, we found significant improvements in the diagnostic efficacy of nomograms compared with imaging or imaging histology alone, with internal and external validations demonstrating good discrimination and calibration, and decision curve analysis demonstrating clinical utility. In summary, the use of clinical-imaging histogram nomograms to predict small volumes of recurrent laryngeal nerve paralymph node metastasis in esophageal cancer can improve the accuracy of prediction by conventional CT techniques and help clinical decision making.

The nomogram in this study is a linear model based on the principle of logistic regression. Finally, this study includes independent risk factors such as rad-score, differentiation degree, N-staging and peripheral nerve infiltration. Using the joint prediction model, the diagnostic efficiency of the training and test set is higher. The KNN-based radiomics model shows that disease assessment needs to integrate different information such as clinical, pathology, imaging, etc. This study integrates multiple-dimensional prediction factors to build a visual line chart model. The calibration curve display model has good fitting advantages, and the model established in this study has good stability and extrapourability. The AUC of the nomogram were 0.97, 0.86 and 0.63 in the training set, internal test group and external test set respectively.

There are several limitations in this study. First, the number of patients included is limited, and the application of machine learning models to big data sets yields more stable results. Several imaging models, including KNN, MLP, and SVM, were included in this study and are a subset of machine learning models with a high ability to simulate nonlinear characteristic data. However, they did not exhibit the expected predictive power in this study, possibly because variable characteristics were not efficiently extracted and the data volume was small. Therefore, in subsequent studies, more multicenter data can be added for training and external validation, resulting in more reliable prediction models. Secondly, the development and validation of this study using retrospective data should be preceded by a prospective validation study to confirm the reliability of the model before formal clinical practice.

## Conclusion

The comprehensive nomogram based on CT images is useful in predicting recurrent laryngeal nerve paranodal lymph node metastasis in patients with esophageal squamous cell carcinoma. The reliable and predictive model can help clinicians to tailor their diagnosis and treatment, improve outcomes and improve postoperative quality of life. Subsequent studies are required to confirm these findings.

## Methodology

- Retrospective,
- Case–control study,
- Performed at two institution.

### Supplementary Information

**Additional file 1: Figure S1.** Schematics to illustrate the extent of observation of paralaryngeal lymph nodes that can be detected by CT examination. **Figure S2.** a The most valuable features were screened out by tuning Lambda using LASSO via minimum binomial deviation. LASSO, least absolute shrinkage and selection operator. b LASSO coefficient profile plot with different log (λ) was shown. The vertical dashed lines represent 19 radiomics features with nonzero coefficients selected with the optimal Lambda value. **Figure S3.** An example of the nomogram in clinical utility. The figure illustrates the process of calculating the risk scores of recurrent laryngeal nerve invasion in ESCC using the nomogram. This is an example of a 70-year-old man with cancer. His recurrent laryngeal nerve mutation Risk score was calculated as 0.4 according to the formula for the Radscore. The total score was 135, which corresponded to a KRAS mutation risk of 0.85. The normal range for the length of esophageal resection is less than 5 cm. The normal range of clinical data is “0”, and the abnormal range is “1”. **Figure S4.** The combined model calibration curve in the training (a) and test (b) cohort, illustrating the relationship between Mean Predicted Probability on the x-axis and Fraction of Positive on the y-axis, while comparing their alignment with the perfectly calibrated line. Rad, radiomics model (KNN). **Figure S5.** DCA curve of radscore and the significantly associated clinical features. **Figure S6.** An example of the nomogram in clinical utility. In this patient, postoperative pathology was confirmed to be differentiation-esophageal squamous cell-peripheral nerve invasion positive, and two small lymph nodes were found on the left clavicle at preoperative CT with clear borders. The probability of metastatic involvement of the paralaryngeal lymph nodes was greater than 75% after calculation of the clinical-imaging nomogram described above, but postoperative pathology of these two lymph nodes alone showed that the two lymph nodes were negative.

## Data Availability

The datasets used and/or analyzed during the current study are available from the TCIA (https://www.cancerimagingarchive.net) databases.

## References

[1] Bray F, Ferlay J, Soerjomataram I (2018). Global cancer statistics 2018: GLOBOCAN estimates of incidence and mortality worldwide for 36 cancers in 185 countries. CA Cancer J Clin.

[2] Wei W, Chen Z, He Y (2015). Long-term follow-up of a community assignment, one-time endoscopic screening study of esophageal cancer in China. J Clin Oncol.

[3] Kanemura T, Makino T, Miyazaki Y (2017). Distribution patterns of metastases in recurrent laryngeal nerve lymph nodes in patients with squamous cell esophageal cancer. Dis Esophagus.

[4] Nakagawa S, Nishimaki T, Kosugi S (2003). Cervical lymphadenectomyis beneficial for patients with carcinoma of the upper and mid-thoracic esophagus. Dis Esophagus.

[5] Igaki H, Tachimori Y, Kato H (2004). Improved survival for patients with upper and/or middle mediastinal lymph node metastasis of squamous cell carcinoma of the lower thoracic esophagus treated with 3-field dissection. Ann Surg.

[6] Ma GW, Situ DR, Ma QL (2014). Three-field vs two-field lymphnode dissection for esophageal cancer: a meta-analysis. World J Gastroenterol.

[7] Jiang K, Huang H, Chen W (2021). Risk factors for lymph node metastasis in T1 esophageal squamous cell carcinoma: a systematic review and meta-analysis. World J Gastroenterol.

[8] Kumakura Y, Yokobori T, Yoshida T (2018). Elucidation of the anatomical mechanism of nodal skip metastasis in superficial thoracic esophageal squamous cell carcinoma. Ann Surg Oncol.

[9] Mazurowski A (2015). Radiogenomics: what it is and why it is important. J Am Coll Radiol.

[10] Yang L, Dong D, Fang M (2018). Can CT-based radiomics signature predict KRAS/NRAS/BRAF mutations in colorectal cancer. Eur Radiol.

[11] Dhruv B, Mittal N, Modi M (2019). Study of Haralick’s and GLCM texture analysis on 3D medical images. Int J Neurosci.

[12] Li Y, Yu M, Wang G (2021). Contrast-enhanced CT-based radiomics analysis in predicting lymphovascular invasion in esophageal squamous cell carcinoma. Front Oncol.

[13] Tang S, Ou J, Liu J (2021). Application of contrast-enhanced CT radiomics in prediction of early recurrence of locally advanced oesophageal squamous cell carcinoma after trimodal therapy. Cancer Imaging.

[14] Luo H, Chen Y, Huang W (2021). Development and validation of a radiomics-based model to predict local progression-free survival after chemo-radiotherapy in patients with esophageal squamous cell cancer. Radiat Oncol.

[15] Foley K, Findlay J, Goh V (2018). Novel imaging techniques in staging oesophageal cancer. Best Pract Res Clin Gastroenterol.

[16] Van R, Van L, Lips I, Meijer G (2015). Imaging of oesophageal cancer with FDG-PET/CT and MRI. Clin Radiol.

[17] Ruan R, Chen S, Tao Y (2021). A Nomogram for predicting lymphovascular invasion in superficial esophageal squamous cell carcinoma. Front Oncol.

[18] Yang L, Yang J, Zhou X (2019). Development of a radiomics nomogram based on the 2D and 3D CT features to predict the survival of non-small cell lung cancer patients. Eur Radiol.

[19] Rizzo S, Botta F, Raimondi S (2018). Radiomics: the facts and the challenges of image analysis. Eur Radiol Exp.

[20] Lambin P, Rios-Velazquez E, Leijenaar R (2012). Radiomics: extracting more information from medical images using advanced feature analysis. Eur J Cancer.

[21] Yu YF, Tan YJ, Xie CM (2020). Development and validation of a preoperative magnetic resonance imaging radiomics-based signature to predict axillary lymph node metastasis and disease-free survival in patients with early-stage breast cancer. JAMA Netw Open.

[22] Tan HN, Gan FW, Wu YP (2020). Preoperative prediction of axillary lymph node metastasis in breast carcinoma using radiomics features based on the fat-suppressed T2 sequence. Acad Radiol.

[23] Shan YN, Xu W, Wang R (2020). A nomogram combined radiomics and kinetic curve pattern as imaging biomarker for detecting metastatic axillary lymph node in invasive breast cancer. Front Oncol.

