# Supplementary material for: Potential value of CT-based comprehensive nomogram in predicting occult lymph node metastasis of esophageal squamous cell paralaryngeal nerves: a two-center study
Source: J Transl Med. 2024 Apr 30;22:399. doi: 10.1186/s12967-024-05217-4 (PMC11059581; doi:10.1186/s12967-024-05217-4)
Supplement: Supplementary file 1 — Additional file 1: Figure S1. Schematics to illustrate the extent of observation of paralaryngeal lymph nodes that can be detected by CT examination. Figure S2. a The most valuable features were screened out by tuning Lambda using LASSO via minimum binomial deviation. LASSO, least absolute shrinkage and selection operator. b LASSO coefficient profile plot with different log (λ) was shown. The vertical dashed lines represent 19 radiomics features with nonzero coefficients selected with the optimal Lambda value. Figure S3. An example of the nomogram in clinical utility. The figure illustrates the process of calculating the risk scores of recurrent laryngeal nerve invasion in ESCC using the nomogram. This is an example of a 70-year-old man with cancer. His recurrent laryngeal nerve mutation Risk score was calculated as 0.4 according to the formula for the Radscore. The total score was 135, which corresponded to a KRAS mutation risk of 0.85. The normal range for the length of esophageal resection is less than 5 cm. The normal range of clinical data is “0”, and the abnormal range is “1”. Figure S4. The combined model calibration curve in the training (a) and test (b) cohort, illustrating the relationship between Mean Predicted Probability on the x-axis and Fraction of Positive on the y-axis, while comparing their alignment with the perfectly calibrated line. Rad, radiomics model (KNN). Figure S5. DCA curve of radscore and the significantly associated clinical features. Figure S6. An example of the nomogram in clinical utility. In this patient, postoperative pathology was confirmed to be differentiation-esophageal squamous cell-peripheral nerve invasion positive, and two small lymph nodes were found on the left clavicle at preoperative CT with clear borders. The probability of metastatic involvement of the paralaryngeal lymph nodes was greater than 75% after calculation of the clinical-imaging nomogram described above, but postoperative pathology of these two lymph nodes [file 12967_2024_5217_MOESM1_ESM.docx]

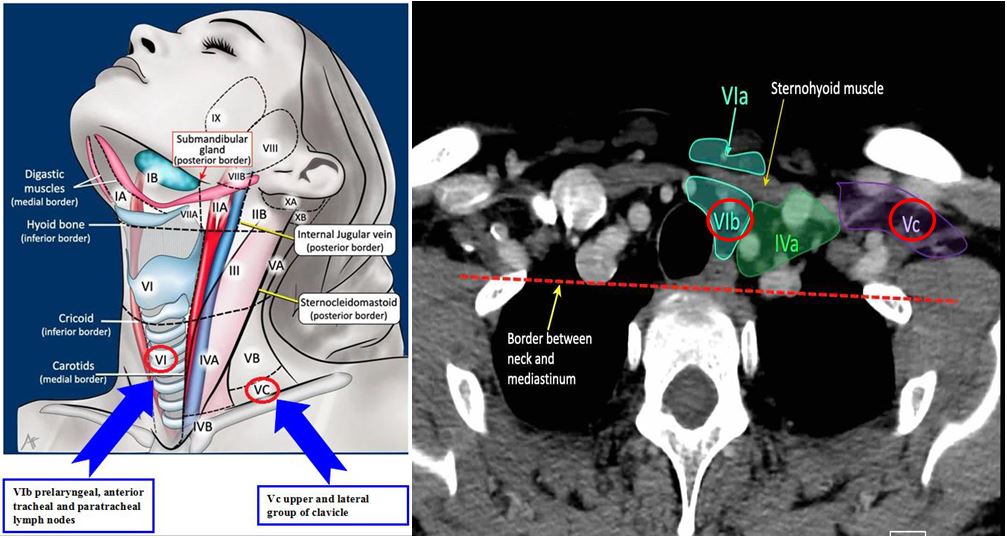


**Fig. S1.** Schematics to illustrate the extent of observation of paralaryngeal lymph nodes that can be detected by CT examination.


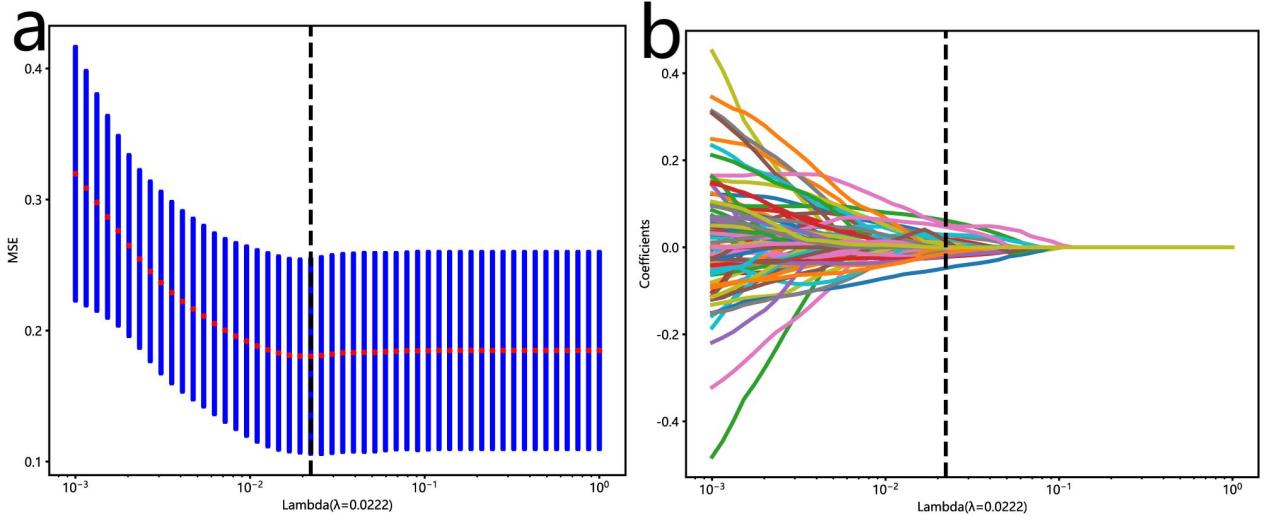


**Fig. S2.** a The most valuable features were screened out by tuning Lambda using LASSO via minimum binomial deviation. LASSO, least absolute shrinkage and selection operator. b LASSO coefficient profile plot with different log (λ) was shown. The vertical dashed lines represent 19 radiomics features with nonzero coefficients selected with the optimal Lambda value.


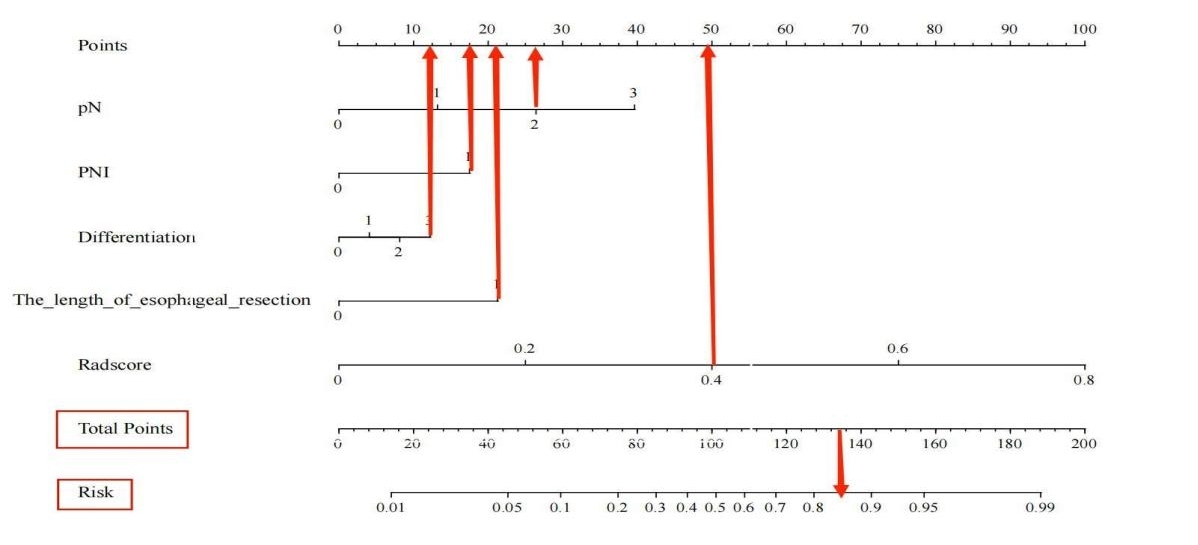


**Fig. S3.** An example of the nomogram in clinical utility. The figure illustrates the process of calculating the risk scores of recurrent laryngeal nerve invasion in ESCC using the nomogram. This is an example of a 70-year-old man with cancer. His recurrent laryngeal nerve mutation Risk score was calculated as 0.4 according to the formula for the Radscore. The total score was 135, which corresponded to a KRAS mutation risk of 0.85.

Note: The normal range for the length of esophageal resection is less than 5cm. The normal range of clinical data is “0”, and the abnormal range is “1”.


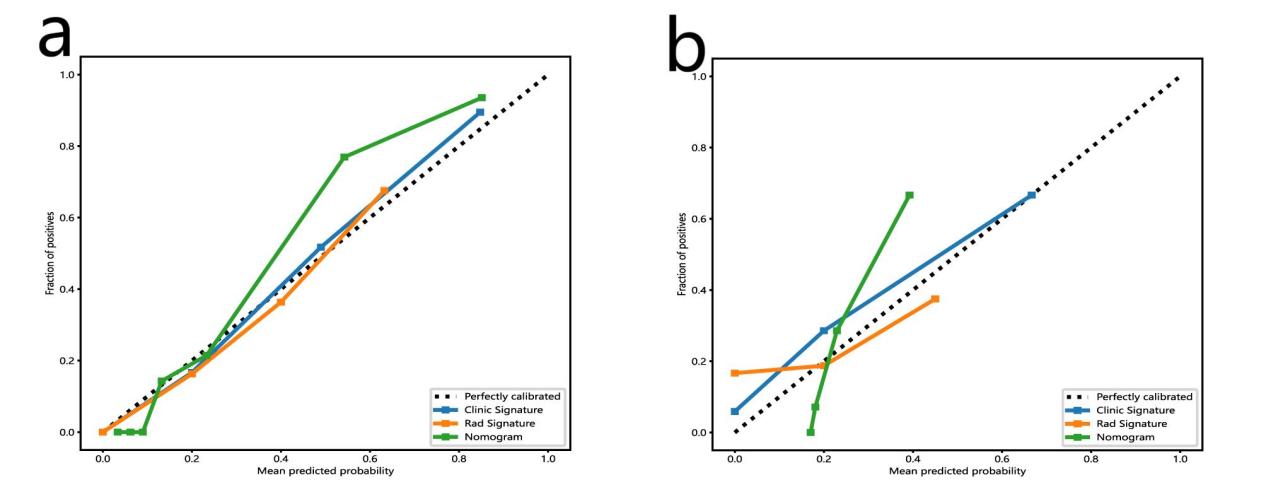


**Fig. S4.** The combined model calibration curve in the training (a) and test (b) cohort, illustrating the relationship between Mean Predicted Probability on the x-axis and Fraction of Positive on the y-axis, while comparing their alignment with the perfectly calibrated line. Rad, radiomics model (KNN).


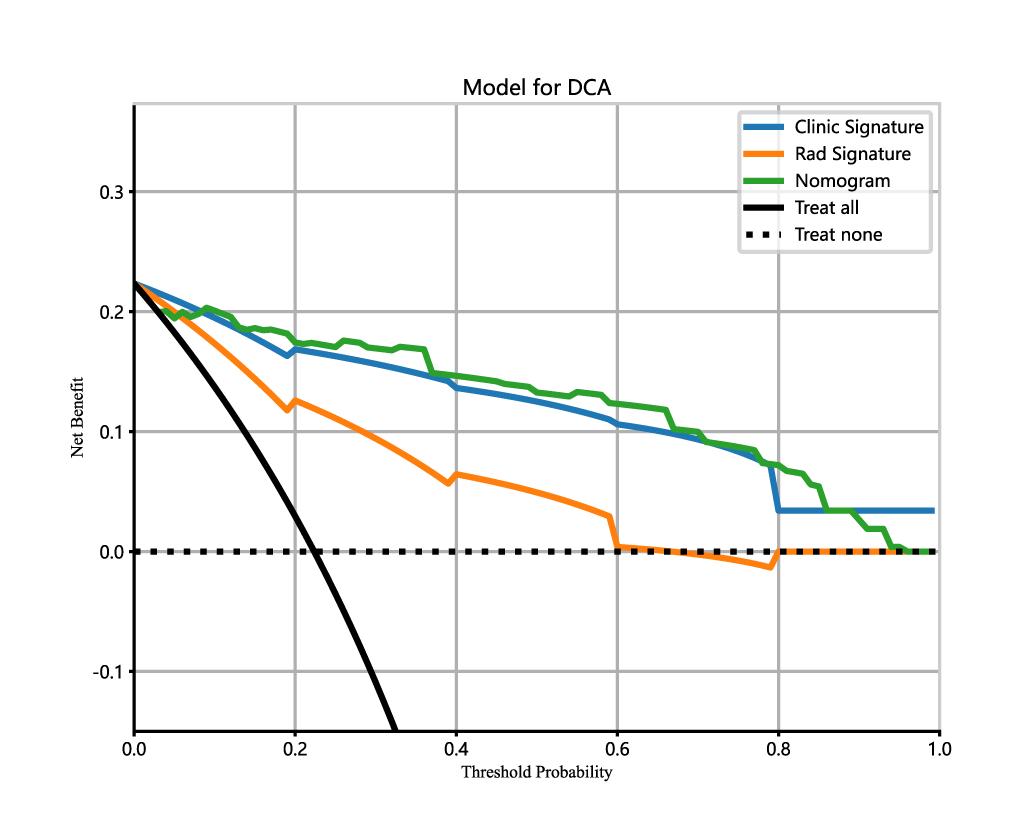


**Fig. S5.** DCA curve of radscore and the significantly associated clinical features.


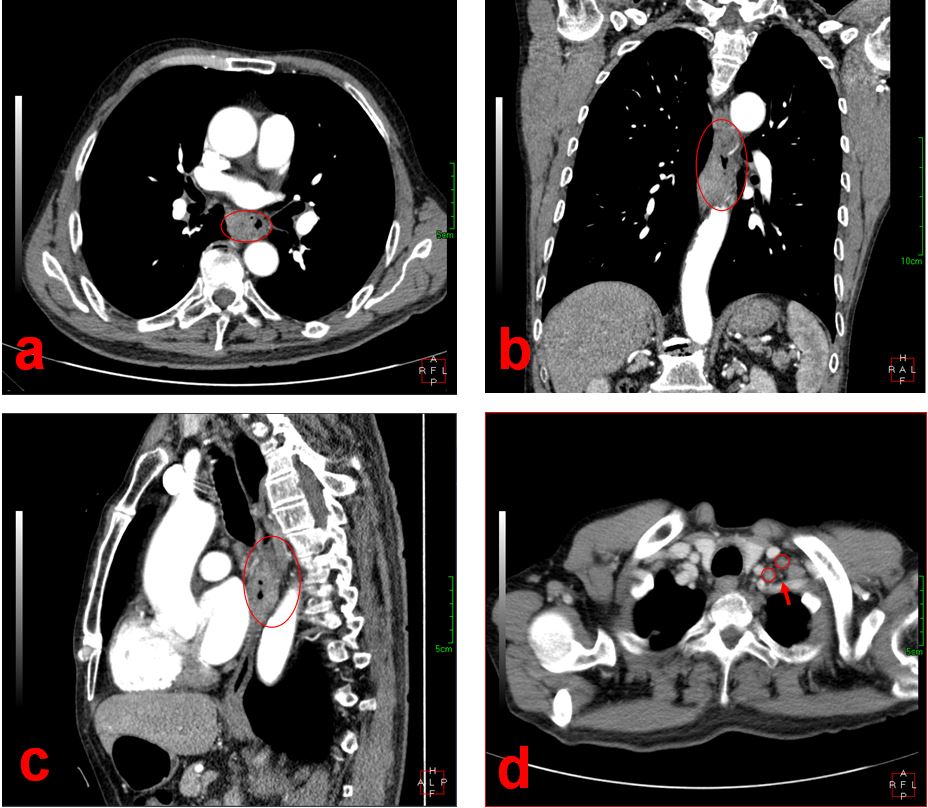


**Fig. S6.** An example of the nomogram in clinical utility. In this patient, postoperative pathology was confirmed to be differentiation-esophageal squamous cell-peripheral nerve invasion positive, and two small lymph nodes were found on the left clavicle at preoperative CT with clear borders. The probability of metastatic involvement of the paralaryngeal lymph nodes was greater than 75% after calculation of the clinical-imaging nomogram described above, but postoperative pathology of these two lymph nodes alone showed that the two lymph nodes were negative.
